# A Hybrid Sliding Window Optimizer for Tightly-Coupled Vision-Aided Inertial Navigation System

**DOI:** 10.3390/s19153418

**Published:** 2019-08-04

**Authors:** Junxiang Jiang, Xiaoji Niu, Ruonan Guo, Jingnan Liu

**Affiliations:** 1 GNSS Research Center, Wuhan University, No. 129 Luoyu Road, Wuhan 430079, China; 2 Collaborative Innovation Center of Geospatial Technology, Wuhan University, No. 129 Luoyu Road, Wuhan 430079, China; 3 School of Geodesy and Geomatics, Wuhan University, No. 129 Luoyu Road, Wuhan 430079, China

**Keywords:** sliding window, optimization, VINS, MSC, marginalization, conditioning

## Abstract

The fusion of visual and inertial measurements for motion tracking has become prevalent in the robotic community, due to its complementary sensing characteristics, low cost, and small space requirements. This fusion task is known as the vision-aided inertial navigation system problem. We present a novel hybrid sliding window optimizer to achieve information fusion for a tightly-coupled vision-aided inertial navigation system. It possesses the advantages of both the conditioning-based method and the prior-based method. A novel distributed marginalization method was also designed based on the multi-state constraints method with significant efficiency improvement over the traditional method. The performance of the proposed algorithm was evaluated with the publicly available EuRoC datasets and showed competitive results compared with existing algorithms.

## 1. Introduction

Accurate localization in an unknown environment is essential for a robot to succeed in its missions. In many cases, existing external localization systems, such as motion capture systems, the global positioning system, or a pre-constructed map of the working area, are costly, insufficiently accurate, or unavailable. In this study, we focused on estimating a vehicle’s motion by fusing the measurements from a monocular camera and an inertial measurement unit (IMU). This task—the well-known monocular vision-aided inertial navigation system (VINS) problem—has drawn great interest in the robotic community (e.g., [1,2,3,4,5,6,7]) for many reasons.

First, the monocular camera and the micro-electro-mechanical system IMU are both small and cheap and consume little power, and both are passive sensors, which means that they exert no influence on the external environment and do not interfere with each other like Lidars and RGB-D cameras. Second, the monocular camera cannot compute inter-frame motion alone, so some studies have assumed a constant-velocity model [2,3] between two frames. However, this assumption results in an inconsistent estimation. The micro-electro-mechanical system IMU usually works at a higher frequency than the monocular camera, so the IMU measurements can be integrated for accurate recovery of short-term inter-frame motion. However, the IMU measurements are corrupted by noise and slowly time-varying biases, which makes long-time integration unreliable [4,8], so visual information is required to aid the IMU in estimation of biases. Third, the monocular camera is a projective sensor that provides bearing information regarding visual features, so motion and structure can only be recovered up to an unknown scale [1,5]. In order to recover its metric scale by using IMU, a large enough acceleration and rotation rate are required along at least two axes of IMU [9]. The direction of the gravity vector is also observable [4,8,9], which means that the absolute roll and pitch of the VINS do not drift. Fourth, unlike some other sensors, such as wheel odometry and two-dimensional (2D) Lidar, IMUs and cameras both allow for three-dimensional (3D) motion and structure estimation. Hence, the system is versatile and applicable to various platforms, such as micro-aerial vehicles (MAVs), smart-phones, and humanoid robots.

The frameworks for information fusion of the VINS problem found in the literature fall into two categories: the loosely coupled framework [1,10,11] and the tightly-coupled framework [4,8,12,13,14,15,16]. The loosely coupled framework uses the results from a standalone visual system directly for fusion with the IMU measurements. For the monocular visual system, the visual scale is also included into the states of the filter. This kind of framework has some obvious drawbacks. First, there is a scale drift for monocular visual systems [10,17]; the most current estimation of the visual scale is used as the scale of the whole trajectory and structure, and the time-varying nature of the visual scale is not considered and cannot be modeled analytically [10]. Second, the two systems operate independently, and the visual system is not assisted by the IMU measurements, so in this case, the fusion results are not optimal [10,11]. In contrast, the tightly-coupled framework integrates the visual and inertial measurements into one likelihood function, so the fusion is optimal. In this paper, we used the tightly-coupled framework.

The algorithms of information fusion of the VINS problem can also be grouped into two major categories: recursive Bayesian filters [1,9,10,11,12,15,16] and bundle adjustment (BA)/graph-based optimization/smoothing methods [4,8,18]. Bayesian filters are typically required to operate at the frame rate. For real-time performance, Bayesian filters marginalize out past poses and summarize the information gained over time with a joint probability distribution that serves as a prior. The computational cost of propagating joint distributions scales poorly with the dimension of the state vector. Hence, for the filters that model the 3D positions of map points in the map as the elements of the state vector, the number of map points in the map is severely limited, so the fusion precision is deduced, which is the main drawback of the filters [19]. The multistate constraint Kalman filter (MSCKF) is a type of augmented extended Kalman filter [12,15,16], its state vector keeps a sliding window of the past poses. The visual feature measurements are used to construct a probabilistic constraint between the poses. Its 3D positions are not modeled as the elements of the state vector. Hence, the computational complexity of MSCKF is linear with the number of features [12,15] and cubic with the length of the sliding window [16]. For the MSCKF, the number of map points in the map is not limited, and it can achieve better performance when combined with delayed linearization. However, some information is lost if the tracking length of the visual feature is outside the range of the sliding window [16]. The MSCKF and the extended Kalman filter are both susceptible to gradual accumulation of linearization errors. This problem becomes more serious if the number of long-term tracked visual features is small, such as during rapid rotation.

The graph-based optimization/BA methods possess many advantages, such as the iterative re-linearization that makes linearization errors negligible, the batch processing that makes the estimation results optimal and consistent, and the ability to add and remove measurements expediently. Compared with standard numerical optimization, the core features of the graph-based optimization methods take advantage of the sparsity of structure, including first- and second-order sparsity [20,21]. The sparsity makes the graph-based optimization methods particularly efficient. Nevertheless, as information accumulates, a full BA quickly becomes infeasible in computation for real-time or near real-time operation. To keep the computing time bounded, an alternative method is the local BA (LBA) that operates in the spirit of BA but maintains only some poses in the path (typically a sliding window of the most current poses; e.g., [3,4,8,18,21]) and the observable map points. Obviously, direct removal of the edges related with the nodes outside the sliding window of the LBA is unwarranted. The literature includes two main types of methods to cope with those edges: the conditioning-based method [3,4] and the prior-based method [8,18]. For the conditioning-based method, the nodes outside the sliding window are directly fixed, and the related edges are used as usual. After the measurements of IMU are added, the first node (including the pose, velocity, and biases of IMU) of the sliding window must also be fixed to eliminate the ambiguity of motion [4]. This kind of method is highly robust but is not optimal theoretically, because only a part of the graph is active while LBA is performed. For the prior-based method, a marginalization technique is performed on the edges related with the outside nodes to construct a prior distribution (typically, a Gaussian distribution) for the nodes in the sliding window. This kind of method is optimal theoretically but is also affected by linearization errors numerically and cannot cope properly with features whose tracking length lies outside the range of the sliding window.

In this paper, we propose a novel hybrid sliding window optimizer (HSWO) that has the advantages of both the conditioning-based and prior-based methods. To make the linearization points of the prior distribution reliable, the sliding window in our method is divided into two parts. We call the front part the mature region and the back part the growing region. We marginalize a map point out only if its last measurement lies within the mature region. To cope with map points whose tracking length lies outside the range of the sliding window, we follow the conditioning-based method. The nodes outside the sliding window are fixed directly and are called the fixed basis. For balancing the linearization errors within the growing region and fixed basis, the size of the mature region should be selected carefully.

The two kinds of marginalization techniques—the Schur complement technology [8,18] and the null-space-based method [12,15,16]—are equivalent mathematically [22]. For the traditional method, all reprojection factors of marginalized map points are linearized at the current estimation, and a Hessian matrix is constructed by stacking linearized factors. Because there is a submatrix in the Hessian matrix that needs to be inversed, and the dimension of the submatrix depends on the number of marginalized map points (possibly more than hundreds). It is time-consuming and numerically unstable if we directly calculate the inverse matrix of the submatrix, as in [8]. For our method, we first used the null-space-based method to calculate the multi-state constraints factors of the marginalized map points, i.e., the map point position parameters were marginalized first. Then, we stacked the multistate constraint factors to construct the Hessian matrix. In our method, the dimension of the submatrix that needs to be inversed is quite low.

To avoid the repeated integration of IMU measurements, the well-known IMU pre-integration technology has been widely adopted in graph-based optimization methods. This technique was first proposed by Lupton et al. [23,24] and further improved by [8,25,26]. The integration is performed in the first body reference frame during a period, so no previous estimates or covariance are necessary except for the current estimates of the IMU biases.

It is well known that the uncertainty of low-parallax features is poorly approximated by Gaussian distribution in Euclidean (*XYZ*) space, which makes *XYZ* parameterization suitable only for relatively close features. An inverse depth parameterization technology was proposed by Civera et al. to handle this case [5,27]. However, the dimension of the inverse depth parameterization is six, which is twice the size of the *XYZ* parameterization. For this reason, an anchored inverse depth parameterization was proposed by Pietzsch [28]. In this study, we adopted the anchored inverse depth parameterization and selected the first keyframe in the sliding window as the anchored frame.

Some VINS solutions, such as VI-ORB-SLAM [4], assume that the camera-to-IMU transformation is known. This requirement is not always met, such as with a new device. Although the camera-to-IMU transformation can be calibrated by offline methods [29,30], these methods are time-consuming and complex and require a professional user to carefully move the device in front of a stationary calibration target [29,30]. In this study, the camera-to-IMU transformation was estimated online.

Both the conditioning-based method and the prior-based method are susceptible to linearization error. Directly fixing the outside nodes makes the conditioning-based method more sensitive to linearization error, so a high-precision initialization method is essential for the VINS problem [7]. Two types of online initialization methods are found in the literature: loosely-coupled methods [31,32,33] and tightly-coupled methods [34,35]. As reported by Liu et al. [32], the tightly-coupled methods that aim to recover all navigation quantities in one attempt perform poorly because they attempt to solve a large number of variables in a poorly conditioned system. In this study, we adopted the method proposed by Huang et al. [31].

Our contributions are threefold:

- We designed a novel hybrid sliding window optimizer that has the advantages of both the conditioning-based method and the prior-based method.
- We designed a distributed marginalization technology based on multi-state constraint factors.
- We estimated the camera-to-IMU transformation online.

The remainder of this paper is organized as follows. Section 2 briefly introduces the anchored inverse depth parameterization and IMU pre-integration technology. In Section 3, we demonstrate the framework of the hybrid sliding window optimizer in detail. In Section 4, we demonstrate the distributed marginalization technology, and in Section 5, we show our experimental results and some implementation details, and in Section 6, we make our conclusions.

## 2. Measurement Model Formulation

This section briefly introduces the anchored inverse depth parameterization and the IMU pre-integration technology. The anchored inverse depth parameterization effectively improves the accuracy of linearization relative to Euclidean *XYZ* parameterization [5,27]. To avoid the repeated integration of IMU measurements during optimization iteration, IMU pre-integration technology was proposed by Lupton et al. [23,24] and further improved in other studies [8,25,26]. In this study, we followed the one proposed by Qin et al. [8].

### 2.1. Notation and Frames of Reference

In this paper, the world reference frame is denoted as W; the camera reference frame is denoted as C; the IMU body reference frame is denoted as B. All vectors and matrixes are bold, and a vector projected in a specific reference frame is appended with a right superscript, e.g., $p^{B}\in {\mathbb{R}}^{3}$ means the vector p is projected into the reference frame B. A transformation matrix $T_{WB}\in SE^{3}$ which transforms the vector $p^{B}\in {\mathbb{R}}^{3}$ from the reference frame B to the reference frame W. The transformation matrix can be further divided into a rotation matrix $R_{WB}\in SO^{3}$ and a relative translation vector $p_{WB}^{W}\in {\mathbb{R}}^{3}$, as follows:

(1) $$\left[\begin{array}{c} p^{W}\\ 1 \end{array}\right]=T_{WB}\left[\begin{array}{c} p^{B}\\ 1 \end{array}\right]=\left[\begin{array}{cc} R_{WB} & p_{WB}^{W}\\ 0 & 1 \end{array}\right]\left[\begin{array}{c} p^{B}\\ 1 \end{array}\right]$$

In addition, the notation ${\bar{q}}_{WB}$ signifies the unit quaternion corresponding to the rotation matrix $R_{WB}$. We refer readers to [31] for more details about the operation of unit quaternion. The function $T_{WB}(\cdot )$ is defined to transform a vector projected in the reference B to the reference frame W, i.e., $p^{W}=T_{WB}(p^{B})$.

The pose of the IMU at time $t_{k}$ with respect to the world reference frame W is denoted as $T_{WB_{k}}$; the camera-to-IMU transformation is denoted as $T_{BC}$. For a full-rank square matrix A, the notation $A^{-1}$ signifies the inverse matrix of A, i.e., $A^{-1}A=AA^{-1}=I$. The notation I signifies identity matrix. The notation $A^{T}$ signifies the transposed matrix of A. For a vector $p={\left[p_{x},p_{y},p_{z}\right]}^{T}\in {\mathbb{R}}^{3}$, the notation ${\left[p\right]}_{\times }$ signifies the skew-symmetric matrix of p, as follows:

(2) $${\left[p\right]}_{\times }=\left[\begin{array}{ccc} 0 & -p_{z} & p_{y}\\ p_{z} & 0 & -p_{x}\\ -p_{y} & p_{x} & 0 \end{array}\right]$$

### 2.2. Inverse Depth Parameterization

Assume that the map point $l_{m}$ is co-visible in the keyframe $KF_{a}$ and $KF_{j}$. We selected $KF_{a}$ as the anchored keyframe for $l_{m}$. The pose of the anchored keyframe is denoted as $T_{WB_{a}}$. The pose of the observing keyframe $KF_{j}$ is denoted as $T_{WB_{j}}$. Let $\psi^{l_{m}}={\left[\psi_{1},\psi_{2},\psi_{3}\right]}^{T}\in {\mathbb{R}}^{3}$ be the inverse depth parameterization of the co-visible map point in the anchored camera reference frame $C_{a}$. The function $\Pi (\psi^{l_{m}})={\left[\frac{\psi_{1}}{\psi_{3}},\;\frac{\psi_{2}}{\psi_{3}},\frac{1}{\psi_{3}}\right]}^{T}={\left[X^{C_{a}},Y^{C_{a}},Z^{C_{a}}\right]}^{T}$ transforms an inverse depth parameterization to its Euclidean *XYZ* counterpart and vice versa. We adopted a conventional pinhole-camera model, and the 2D projection of the co-visible map point on the image plane of the anchored keyframe are

(3) $$z_{a}^{l_{m}}(\psi^{l_{m}})=\left[\begin{array}{c} f_{u}\frac{X^{C_{a}}}{Z^{C_{a}}}+u_{0}\\ f_{v}\frac{Y^{C_{a}}}{Z^{C_{a}}}+v_{0} \end{array}\right]=\left[\begin{array}{c} f_{u}\psi_{1}+u_{0}\\ f_{v}\psi_{2}+v_{0} \end{array}\right]$$

where ${\left[f_{u}\;f_{v}\right]}^{T}$ is the focal length, and ${\left[u_{0}\;v_{0}\right]}^{T}$ is the principal point. 

The *XYZ* coordinates of the co-visible map point $l_{m}$ in the observing camera reference frame $C_{j}$ are

(4) $${\left[X^{C_{j}},Y^{C_{j}},Z^{C_{j}}\right]}^{T}=T_{BC}^{-1}T_{WB_{j}}^{-1}T_{WB_{a}}T_{BC}\left(\Pi (\psi^{l_{m}})\right)$$

the 2D projection of the co-visible map point $l_{m}$ on the image plane of the observing keyframe are

(5) $$z_{j}^{l_{m}}(\psi^{l_{m}},T_{WB_{a}},T_{WB_{j}},T_{BC})=\left[\begin{array}{c} f_{u}\frac{X^{C_{j}}}{Z^{C_{j}}}+u_{0}\\ f_{v}\frac{Y^{C_{j}}}{Z^{C_{j}}}+v_{0} \end{array}\right]$$

this model does not consider the distortion of the camera lens. All 2D coordinates of the key points are undistorted immediately after extraction.

Let ${\tilde{z}}_{a}^{l_{m}}$ and ${\tilde{z}}_{j}^{l_{m}}$ be the 2D positions of the key points in the anchored keyframe and the observing keyframe, respectively, and their extraction errors $\eta_{a}^{l_{m}}$ and $\eta_{j}^{l_{m}}$ are typically zero-mean Gaussian. The visual reprojection residual functions are as follows:

(6) $$r_{a}^{l_{m}}={\tilde{z}}_{a}^{l_{m}}-z_{a}^{l_{m}}+\eta_{a}^{l_{m}},\;r_{j}^{l_{m}}={\tilde{z}}_{j}^{l_{m}}-z_{j}^{l_{m}}+\eta_{j}^{l_{m}}$$

where the covariance matrix of $\eta_{a}^{l_{m}}$ and $\eta_{j}^{l_{m}}$ are denoted as $P_{a}^{l_{m}}$ and $P_{j}^{l_{m}}$ (the covariance notation P is capital for making a distinction with the translation notation p which is lowercase).

### 2.3. IMU Pre-Integration Technology

The IMU measures the acceleration $a^{B}$ and the angular velocity $\omega^{B}$ of a vehicle, typically at hundreds of Hz. Both measurements are corrupted by additive measurement noises and time-varying biases; therefore, the raw measurements of IMU at time t are modeled as follows:

(7) $${\tilde{a}}^{B_{t}}=a^{B_{t}}+b_{a}^{B_{t}}+n_{a}^{B_{t}},\;{\tilde{\omega }}^{B_{t}}=\omega^{B_{t}}+b_{g}^{B_{t}}+n_{g}^{B_{t}}$$

where the notation $b_{\left(\cdot \right)}^{B_{t}}$ signifies the time-varying biases of IMU. The notation $n_{\left(\cdot \right)}^{B_{t}}$ signifies the additive noises of IMU and are typically Gaussian, i.e., $n_{a}^{B_{t}}\sim N(0,\sigma_{a}^{2})$, $n_{g}^{B_{t}}\sim N(0,\sigma_{g}^{2})$. The time-varying biases are modeled as a random walk,

(8) $${\dot{b}}_{a}^{B_{t}}=n_{b_{a}}^{B_{t}},\;{\dot{b}}_{g}^{B_{t}}=n_{b_{g}}^{B_{t}}$$

where the noises that drive the biases are Gaussian, i.e., $n_{b_{a}}^{B_{t}}\sim N(0,\sigma_{b_{a}}^{2})$, $n_{b_{g}}^{B_{t}}\sim N(0,\sigma_{b_{g}}^{2})$.

Given two consecutive keyframes at times $t_{k}$ and $t_{k+1}$, all IMU measurements are pre-integrated into a single measurement that serves as a constraint between the IMU poses at times $t_{k}$ and $t_{k+1}$. According to Newton’s second law, the related integration equations are as follows:

(9) $$\begin{array}{ll} p_{WB_{k+1}}^{W} & =p_{WB_{k}}^{W}+v_{WB_{k}}^{W}\Delta t_{k}+\frac{1}{2}g^{W}\Delta t_{k}^{2}+R_{WB_{k}}\iint_{t\in [t_{k},t_{k+1}]}[R_{B_{k}B_{t}}({\tilde{a}}^{B_{t}}-b_{a}^{B_{t}}-n_{a}^{B_{t}})]dt^{2}\\ & =p_{WB_{k}}^{W}+v_{WB_{k}}^{W}\Delta t_{k}+\frac{1}{2}g^{W}\Delta t_{k}^{2}+R_{WB_{k}}\alpha_{B_{k}B_{k+1}}\\ v_{WB_{k+1}}^{W} & =v_{WB_{k}}^{W}+g^{W}\Delta t_{k}+R_{WB_{k}}\int_{t\in [t_{k},t_{k+1}]}[R_{B_{k}B_{t}}({\tilde{a}}^{B_{t}}-b_{a}^{B_{t}}-n_{a}^{B_{t}})]dt\\ & =v_{WB_{k}}^{W}+g^{W}\Delta t_{k}+R_{WB_{k}}\beta_{B_{k}B_{k+1}}\\ {\bar{q}}_{WB_{k+1}} & ={\bar{q}}_{WB_{k}}⊗\int_{t\in [t_{k},t_{k+1}]}\frac{1}{2}[{\tilde{\omega }}^{B_{t}}-b_{g}^{B_{t}}-n_{g}^{B_{t}}]⊗{\bar{q}}_{WB_{t}}dt\\ & ={\bar{q}}_{WB_{k}}⊗\gamma_{B_{k}B_{k+1}} \end{array}$$

where $\Delta t_{k}=t_{k+1}-t_{k}$; ⊗ represents quaternion multiplication, and we refer readers to [31] for more details; $v_{WB_{k}}^{W}$ and $v_{WB_{k+1}}^{W}$ represent the velocity of IMU at time $t_{k}$ and $t_{k+1}$, respectively; $\alpha_{B_{k}B_{k+1}}$, $\beta_{B_{k}B_{k+1}}$, and $\gamma_{B_{k}B_{k+1}}$ are the so-called pre-integrations of the IMU measurements during $\left[t_{k},t_{k+1}\right]$. Note that the IMU pre-integration is performed in the first IMU reference frame $B_{k}$ without the need for initial pose or velocity estimates. Because of the presence of random noises, the pre-integration also involves random variables. We need to calculate its covariance for information fusion. We directly cite the linearized continuous-time dynamic model of the deviation of the pre-integration and refer readers to the literature [8] for more details, as follows:

(10) $$\begin{array}{ll} \left[\begin{array}{l} \delta {\dot{\alpha }}_{B_{k}B_{t}}\\ \delta {\dot{\beta }}_{B_{k}B_{t}}\\ \delta {\dot{\theta }}_{B_{k}B_{t}}\\ \delta {\dot{b}}_{a}^{B_{t}}\\ \delta {\dot{b}}_{g}^{B_{t}} \end{array}\right] & =\left[\begin{array}{ccccc} 0 & I & 0 & 0 & 0\\ 0 & 0 & -R_{B_{k}B_{t}}{\left[{\tilde{a}}^{B_{t}}-{\hat{b}}_{a}^{B_{t}}\right]}_{\times } & -R_{B_{k}B_{t}} & 0\\ 0 & 0 & -{\left[{\tilde{\omega }}^{B_{t}}-{\hat{b}}_{g}^{B_{t}}\right]}_{\times } & 0 & -I\\ 0 & 0 & 0 & 0 & 0\\ 0 & 0 & 0 & 0 & 0 \end{array}\right]\left[\begin{array}{l} \delta \alpha_{B_{k}B_{t}}\\ \delta \beta_{B_{k}B_{t}}\\ \delta \theta_{B_{k}B_{t}}\\ \delta b_{a}^{B_{t}}\\ \delta b_{g}^{B_{t}} \end{array}\right]+\left[\begin{array}{cccc} 0 & 0 & 0 & 0\\ -R_{B_{k}B_{t}} & 0 & 0 & 0\\ 0 & -I & 0 & 0\\ 0 & 0 & I & 0\\ 0 & 0 & 0 & I \end{array}\right]\left[\begin{array}{c} n_{a}^{B_{t}}\\ n_{g}^{B_{t}}\\ n_{b_{a}}^{B_{t}}\\ n_{b_{g}}^{B_{t}} \end{array}\right]\\ & =A_{t}\delta {\overset{˘}{x}}_{t}+B_{t}n_{t},t\in [t_{k},t_{k+1}] \end{array}$$

where $\delta {\bar{q}}_{B_{k}B_{t}}=[1\frac{1}{2}\delta \theta_{B_{k}B_{t}}^{T}{]}^{T}$ represents the orientation error.

According to the linear system theorem [36], this system can be discretized as follows:

(11) $$\begin{array}{ll} \delta {\overset{˘}{x}}_{t+\Delta T} & \approx (I+\Delta TA_{t})\delta {\overset{˘}{x}}_{t}+\Delta TB_{t}{\bar{n}}_{t}\\ & ≐\Phi_{t+\Delta T,t}\delta {\overset{˘}{x}}_{t}+G_{t}{\bar{n}}_{t} \end{array}$$

where ΔT is the sampling time of IMU; ${\bar{n}}_{t}=\frac{1}{\Delta T}\int_{t}^{t+\Delta T}n_{\tau }d\tau$ is the equivalent noise during [t,t+ΔT]; its mean is zero, and its covariance is calculated as follows:

(12) $$\mathrm{cov}({\bar{n}}_{t})=\frac{1}{\Delta T^{2}}\int_{t}^{t+\Delta T}E(n_{\tau }n_{\tau }^{T})d\tau =\frac{1}{\Delta T}\mathrm{cov}(n_{t})=\frac{Q}{\Delta T}=\bar{Q}$$

where $Q=\mathrm{diag}(\sigma_{a}^{2},\sigma_{g}^{2},\sigma_{b_{a}}^{2},\sigma_{b_{g}}^{2})$. Because ${\bar{n}}_{t}$ is a Gaussian white noise sequence, the covariance of the deviation of the pre-integration can be calculated recursively, as follows:

(13) $$P_{t+\Delta T}=\Phi_{t+\Delta T,t}P_{t}\Phi_{t+\Delta T,t}^{T}+G_{t}\bar{Q}G_{t}^{T}$$

where the initial covariance $P_{k}$ is set to be zeros.

The residual function about the IMU pre-integration in $\left[t_{k},t_{k+1}\right]$ is

(14) $$r_{IMU}^{k+1,k}=\left[\begin{array}{c} p_{WB_{k+1}}^{W}-p_{WB_{k}}^{W}-v_{WB_{k}}^{W}\Delta t_{k}-\frac{1}{2}g^{W}\Delta t_{k}^{2}-R_{WB_{k}}\alpha_{B_{k}B_{k+1}}(b_{a}^{B_{k}},b_{g}^{B_{k}})\\ v_{WB_{k+1}}^{W}-v_{WB_{k}}^{W}-g^{W}\Delta t_{k}-R_{WB_{k}}\beta_{B_{k}B_{k+1}}(b_{a}^{B_{k}},b_{g}^{B_{k}})\\ {\bar{q}}_{WB_{k+1}}^{-1}⊗{\bar{q}}_{WB_{k}}⊗\gamma_{B_{k}B_{k+1}}(b_{g}^{B_{k}})\\ b_{a}^{B_{k+1}}-b_{a}^{B_{k}}\\ b_{g}^{B_{k+1}}-b_{g}^{B_{k}} \end{array}\right]+n_{pre}$$

where $\mathrm{cov}(n_{pre})=P_{k+1}$.

Note that $\alpha_{B_{k}B_{k+1}}(b_{a}^{B_{k}},b_{g}^{B_{k}})$, $\beta_{B_{k}B_{k+1}}(b_{a}^{B_{k}},b_{g}^{B_{k}})$, and $\gamma_{B_{k}B_{k+1}}(b_{g}^{B_{k}})$ are functions about the IMU biases. To perform iterative optimization, we require their Jacobian matrices about the IMU biases. Therefore, the transfer matrix $\Phi_{k+1,k}$ is computed recursively as follows.

(15) $$\Phi_{t+2\Delta T,t}=\Phi_{t+2\Delta T,t+\Delta T}\Phi_{t+\Delta T,t}$$

the related sub-matrices of $\Phi_{k+1,k}$ are the Jacobian matrices.

## 3. Hybrid Sliding Window Optimizer

The best modern systems work via interleaved tracking and mapping via optimization [21]. One of the most representative works is PTAM [37], which was the first work to split mapping and tracking in parallel threads. Based on the main ideas of PTAM, the famous ORB-SLAM was proposed by Mur-Artal [3], and its inertial version was described by Mur-Artal et al. [4]. For this study, we built our implementation upon the local mapping thread of ORB-SLAM. We claim that the hybrid sliding window optimizer is generic and suitable for any keyframe-based system with a pipeline similar to that of PTAM or ORB-SLAM.

### 3.1. Framework

The hybrid sliding window optimizer is performed once a new keyframe is received. We kept the last N successive keyframes in the sliding window and all map points visible by those N keyframes. Co-visible keyframes outside the sliding window are fixed during optimization.

**Figure 1:**
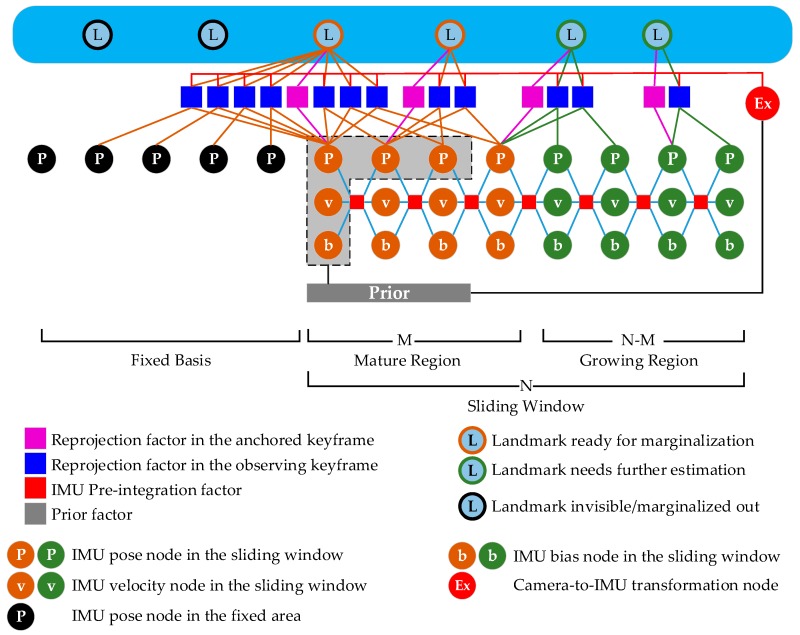
The structure of the hybrid sliding window optimizer.

Figure 1 illustrates the framework of the hybrid sliding window optimizer. The sliding window is divided into two parts: the mature region and the growing region. The nodes (state variables) within the mature region have been updated many times and doubtless possess greater estimation precision than those in the growing region. The map point is to be marginalized out only if all of its edges (at least 3) are related only with the nodes in the mature region (at least 2) and the fixed basis, which decreases the linearization error. As a result, the prior becomes more accurate. After each update, the first IMU pose, velocity, and bias nodes and all 3D position nodes of the map points that meet the above criteria are marginalized out. A prior factor is then constructed that is related to the IMU pose nodes (excluding the first one) within the mature region and the second IMU velocity and bias nodes. Finally, all factors used for marginalization are removed from the sliding window. This process is repeated once a new keyframe is received. For map points with a tracking length greater than the size of the sliding window, we continue to estimate the 3D positions until all edges (residuals) move into the mature region and the fixed basis. Some existing methods, such as MSCKF [12,15] and SWF [8,18], cannot deal well with this kind of map point.

### 3.2. Formulation

In this section, the cost function used for the graph-based optimization is constructed. All related residual functions and state variables are defined in a compact form. Let the full state variables of IMU as $x_{k}={\left[{\bar{q}}_{WB_{k}}^{T},p_{WB_{k}}^{W}{}^{T},v_{WB_{k}}^{W}{}^{T},b_{a}^{B_{k}}{}^{T},b_{g}^{B_{k}}{}^{T}\right]}^{T}$. Let the state variables of camera-to-IMU transformation as $x_{BC}={\left[{\bar{q}}_{BC}^{T},p_{BC}^{B}{}^{T}\right]}^{T}$. For a visible map point $l_{m}$, let $S_{F}$, $S_{M}$, and $S_{G}$ be the sets of observation keyframes corresponding to the fixed basis, the mature region, and the growing region, respectively. The first observation keyframe in the sliding window is selected as the anchored keyframe $KF_{a}$. We denote the full state variables of IMU about $KF_{a}$ as $x_{a}$. According to Equation (6), the cost function about the map point $l_{m}$ is

(16) $$\begin{array}{ll} C^{l_{m}}= & {\left\|r_{a}^{l_{m}}(\psi^{l_{m}})\right\|}_{P_{a}^{l_{m}}}^{2}+\sum_{i\in S_{F}}{\left\|r_{i}^{l_{m}}(x_{a},x_{BC},\psi^{l_{m}})\right\|}_{P_{i}^{l_{m}}}^{2}+\\ & \sum_{j\in \{S_{M}/KF_{a}\}}{\left\|r_{j}^{l_{m}}(x_{a},x_{j},x_{BC},\psi^{l_{m}})\right\|}_{P_{j}^{l_{m}}}^{2}+\sum_{k\in \{S_{G}/KF_{a}\}}{\left\|r_{k}^{l_{m}}(x_{a},x_{k},x_{BC},\psi^{l_{m}})\right\|}_{P_{k}^{l_{m}}}^{2} \end{array}$$

where the visual reprojection residual function $r_{\left(\cdot \right)}^{l_{m}}$ is only related with a part of elements of the full state variables of IMU $x_{\left(\cdot \right)}$, i.e., ${\bar{q}}_{WB_{\left(\cdot \right)}}$ and $p_{WB_{\left(\cdot \right)}}^{W}$. For convenience of notation, we directly used the notation $x_{\left(\cdot \right)}$, the other part of $x_{\left(\cdot \right)}$, i.e., $v_{WB_{\left(\cdot \right)}}^{W}$, $b_{a}^{B_{\left(\cdot \right)}}$, and $b_{g}^{B_{\left(\cdot \right)}}$, has no influence on the $r_{\left(\cdot \right)}^{l_{m}}$. The notation ${\left\|r\right\|}_{P}^{2}$ signifies the Mahalanobis distance of r given the covariance matrix P. The notation C written in “Euclid Math one” signifies the cost function.

According to Equation (14), the cost function about the IMU pre-integration during $\left[t_{k},t_{k+1}\right]$ is

(17) $$C_{IMU}^{k+1}={\left\|r_{IMU}^{k+1,k}(x_{k},x_{k+1})\right\|}_{P_{k+1}}^{2}$$

where $P_{k+1}$ is the covariance matrix of the deviation of the IMU pre-integration, as in Equation (13).

To simplify the notations, the time of the first keyframe in the sliding window is denoted as $t_{0}$. Let $r_{prior}(x_{0},x_{1},\cdots ,x_{M-2})$ be the linearized and normalized prior residual function; its cost function can be written as

(18) $$C_{prior}={\left\|r_{prior}(x_{0},x_{1},\cdots ,x_{M-2})\right\|}_{I}^{2}$$

where M is the size of the mature region.

We stacked the individual state variables into the full state variables of the sliding window, as follows:

(19) $$\chi ={\left[x_{0}^{T},x_{1}^{T},\cdots ,x_{{}_{N-1}}^{T},x_{BC}^{T},\psi^{l_{0}}{}^{T},\cdots ,\psi^{l_{m}}{}^{T},\cdots ,\psi^{l_{K-1}}{}^{T}\right]}^{T}$$

where N denotes the size of the sliding window. K denotes the number of map points visible in the sliding window.

According to Equations (15)–(17), the cost function for optimization is 

(20) $$C(\chi )=C_{prior}+\sum_{m=0}^{K-1}C^{l_{m}}+\sum_{k=0}^{N-1}C_{IMU}^{k+1}$$

The optimal estimation $\chi^{*}$ can be obtained by minimizing the cost function Equation (19):

(21) $$\chi^{*}=\underset{\chi }{\arg\min}C(\chi )$$

The Equation (19) is the cost function of the nonlinear least-squares problem. This problem can be solved with a numerical optimization method [38], such as the Dogleg method, 2D subspace minimization, the Gauss–Newton method, or the Levenberg–Marquardt method. In this study, we adopted the Levenberg–Marquardt method and implemented our optimizer based on Ceres [39].

The likelihood function considers all measurements. To keep the computation time bounded, marginalization technology is used to construct a linearized and normalized prior factor that gains information over time. A fixed basis is used to cope with map points whose tracing length exceeds the size of the sliding window. Both the marginalization technology and the fixed basis introduce linearization error, so the estimation result is suboptimal. If the linearization error is negligible, the estimation result will tend to be optimal.

All nodes within the fixed basis remain constant during optimization iteration. Hence, the structure of the Hessian matrix of the hybrid sliding window optimizer is same as that calculated by traditional sliding window optimizers (e.g., [8,18]), and its sparsity was proven by Sibley et al. [18]. Because of the prior factor and the camera-to-IMU transformation, the second-order sparsity may vanish. As a result, the computation complexity of the hybrid sliding window optimizer is $\max(O(N^{3}),O(N^{2}K))$ [20,21].

## 4. Distributed Marginalization

Marginalization technology is widely used in both Bayesian filters and graph-based optimization methods to make system scalable. For the traditional method (e.g., [8,18]), the last prior residual function, all reprojection residual functions of marginalized map points, and the first pre-integration residual function in the sliding window are directly linearized and normalized at current estimates. Then, all of those linearized and normalized residuals are stacked into one residual (we refer readers to the open-source code of [8] for more details), as follows:

(22) $$\begin{array}{l} r(\overset{˘}{\chi })=\hat{r}(\hat{\overset{˘}{\chi }})+H\delta \overset{˘}{\chi }\\ =\left[\begin{array}{c} {\hat{r}}_{r}\\ {\hat{r}}_{m} \end{array}\right]+\left[\begin{array}{cc} B & E\\ E^{T} & C \end{array}\right]\left[\begin{array}{c} \delta {\overset{˘}{\chi }}_{r}\\ \delta {\overset{˘}{\chi }}_{m} \end{array}\right]=\left[\begin{array}{c} r_{r}\\ r_{m} \end{array}\right] \end{array}$$

where $\overset{˘}{\chi }$ is the state variables related with the residuals used in the marginalization. $\hat{\overset{˘}{\chi }}$ represents the current estimation of $\overset{˘}{\chi }$. The notation $\hat{r}(\hat{\overset{˘}{\chi }})$ denotes the function value of $r(\overset{˘}{\chi })$ at current estimation $\hat{\overset{˘}{\chi }}$. $\delta \overset{˘}{\chi }$ is the perturbation increments of $\overset{˘}{\chi }$ with respect to $\hat{\overset{˘}{\chi }}$, as follows:

(23) $$\begin{array}{ll} \delta \overset{˘}{\chi } & ={\left[\underset{\delta {\overset{˘}{\chi }}_{r}}{\underset{︸}{\delta x_{1}^{T},\cdots ,\delta x_{M-1}^{T},\delta x_{BC}^{T}}},\underset{\delta {\overset{˘}{\chi }}_{m}}{\underset{︸}{\delta x_{0}^{T},\delta \psi^{l_{0}}{}^{T},\delta \psi^{l_{1}}{}^{T},\cdots ,\delta \psi^{l_{K_{M}}}{}^{T}}}\right]}^{T}\\ & =[\delta {\overset{˘}{\chi }}_{r}^{T}\delta {\overset{˘}{\chi }}_{m}^{T}{]}^{T} \end{array}$$

where M is the size of the mature region. $K_{M}$ is the number of marginalized map points. $\delta {\overset{˘}{\chi }}_{m}$ denotes perturbation increments of the state variables that need to be marginalized. $\delta {\overset{˘}{\chi }}_{r}$ corresponds to the state variables that remain. $x_{\left(\cdot \right)}$ is the full state variables of IMU as the above section. The perturbation increments of the elements of $x_{\left(\cdot \right)}$ are additive, e.g., $p_{WB_{\left(\cdot \right)}}^{W}={\hat{p}}_{WB_{\left(\cdot \right)}}^{W}+\delta p_{WB_{\left(\cdot \right)}}^{W}$, except for the unit quaternion ${\bar{q}}_{WB_{\left(\cdot \right)}}$. The perturbation of unit quaternion is defined as follows:

(24) $${\bar{q}}_{WB_{\left(\cdot \right)}}={\hat{\bar{q}}}_{WB_{\left(\cdot \right)}}⊗\delta {\bar{q}}_{WB_{\left(\cdot \right)}}={\hat{\bar{q}}}_{WB_{\left(\cdot \right)}}⊗{\left[1,\frac{1}{2}\delta \theta_{WB_{\left(\cdot \right)}}^{T}\right]}^{T}$$

where $\delta \theta_{WB_{\left(\cdot \right)}}^{T}$ is an angle-axis vector. So, the perturbation increments of $x_{\left(\cdot \right)}$ can be denoted as follows:

(25) $$\delta x_{\left(\cdot \right)}={\left[\delta \theta_{WB_{\left(\cdot \right)}}^{T},{\left(\delta p_{WB_{\left(\cdot \right)}}^{W}\right)}^{T},{\left(\delta v_{WB_{\left(\cdot \right)}}^{W}\right)}^{T},\delta b_{a}^{B_{\left(\cdot \right)}}{}^{T},\delta b_{g}^{B_{\left(\cdot \right)}}{}^{T}\right]}^{T}$$

the perturbation increments of $\psi^{l_{\left(\cdot \right)}}$ are additive, i.e., $\psi^{l_{\left(\cdot \right)}}={\hat{\psi }}^{l_{\left(\cdot \right)}}+\delta \psi^{l_{\left(\cdot \right)}}$.

For calculating the prior residual function for next optimization, the Shur complement technology was used on Equation (21) to marginalize the map points out, yields

(26) $$r_{r}-EC^{-1}r_{m}={\hat{r}}_{r}-EC^{-1}{\hat{r}}_{m}+(B-EC^{-1}E^{T})\delta {\overset{˘}{\chi }}_{r}$$

the Equation (25) is only related with ${\overset{˘}{\chi }}_{r}$, and serves as the prior residual function for next optimization after normalization. The map points that have be marginalized out will never be used again. According to Equation (21), it can be found that the dimension of the submatrix C is $3*K_{M}+15$, and mainly depends on the number of marginalized map points. However, in some cases, the $K_{M}$ will be quite large, e.g., rich texture and fast rotation, which makes the matrix inversion $C^{-1}$ time-consuming and numerically unstable.

In this paper, we designed a distributed marginalization method to reduce the dimension of the submatrix C, in order to make the matrix inversion $C^{-1}$ efficient and stable. The core idea of our method is that we first calculate the MSC factors of the marginalized map points. The 3D inverse depth positions of the map points are marginalized out by using the null-space-based method [12,22]. Therefore, those MSC factors are only related with the poses of IMU within the mature region and the camera-to-IMU transformation.

We marginalized a map point out only if all its measurements (at least 3) lie outside the growing region and at least two measurements lie within the mature region. In mathematic terms, there are $S_{G}=\emptyset$, $\mathrm{card}(S_{M})+\mathrm{card}(S_{F})\geq 3$, and $\mathrm{card}(S_{M})\geq 2$, where card(·) denotes the number of elements of the set. For a marginalized map point, there are no visual measurements within the growing region. According to Equation (15), the cost function of the marginalized map point $l_{m}$ is as follows:

(27) $$\begin{array}{ll} C^{l_{m}} & ={\left\|r_{a}^{l_{m}}(\psi^{l_{m}})\right\|}_{P_{a}^{l_{m}}}^{2}+\sum_{i\in S_{F}}{\left\|r_{i}^{l_{m}}(x_{a},x_{BC},\psi^{l_{m}})\right\|}_{P_{i}^{l_{m}}}^{2}\\ & \;+\sum_{j\in \{S_{M}/KF_{a}\}}{\left\|r_{j}^{l_{m}}(x_{a},x_{j},x_{BC},\psi^{l_{m}})\right\|}_{P_{j}^{l_{m}}}^{2}\\ & ={\left\|{\left[r_{a}^{l_{m}}{\left(\psi^{l_{m}}\right)}^{T}\cdots r_{i}^{l_{m}}{\left(x_{a},x_{BC},\psi^{l_{m}}\right)}^{T}\cdots r_{j}^{l_{m}}{\left(x_{a},x_{j},x_{BC},\psi^{l_{m}}\right)}^{T}\cdots \right]}^{T}\right\|}_{P^{l_{m}}}^{2}\\ & ={\left\|r^{l_{m}}(\tilde{\chi },\psi^{l_{m}})\right\|}_{P^{l_{m}}}^{2} \end{array}$$

where $r^{l_{m}}$ is the reprojection residual function of the map point $l_{m}$. $\tilde{\chi }={\left[\delta x_{0}^{T},\cdots ,\delta x_{M-1}^{T},\delta x_{BC}^{T}\right]}^{T}$ is the state variables including the full state variables of IMU within the mature region and the camera-to-IMU transformation. $P^{l_{m}}=\mathrm{diag}(P_{a}^{l_{m}},\cdots ,P_{i}^{l_{m}},\cdots P_{j}^{l_{m}},\cdots )$ is a block-diagonal covariance matrix. The residual function of $l_{m}$ can be linearized and normalized at the current estimation $\hat{\tilde{\chi }}$ and ${\hat{\psi }}^{l_{m}}$, as follows:

(28) $${\left(P^{l_{m}}\right)}^{-\frac{1}{2}}r^{l_{m}}(\tilde{\chi },\psi^{l_{m}})={\left(P^{l_{m}}\right)}^{-\frac{1}{2}}{\hat{r}}^{l_{m}}(\hat{\tilde{\chi }},{\hat{\psi }}^{l_{m}})+{\left(P^{l_{m}}\right)}^{-\frac{1}{2}}J_{\tilde{\chi }}\delta \tilde{\chi }+{\left(P^{l_{m}}\right)}^{-\frac{1}{2}}J_{\psi }\delta \psi^{l_{m}}$$

where ${\hat{r}}^{l_{m}}(\hat{\tilde{\chi }},{\hat{\psi }}^{l_{m}})$ signifies the function value of $r^{l_{m}}(\tilde{\chi },\psi^{l_{m}})$ at current estimation $\hat{\tilde{\chi }}$ and ${\hat{\psi }}^{l_{m}}$.

We rewrite Equation (28) in a compact form, as follows:

(29) $${\bar{r}}^{l_{m}}(\tilde{\chi },\psi^{l_{m}})={\hat{\bar{r}}}^{l_{m}}(\hat{\tilde{\chi }},{\hat{\psi }}^{l_{m}})+{\bar{J}}_{\tilde{\chi }}\delta \tilde{\chi }+{\bar{J}}_{\psi }\delta \psi^{l_{m}}$$

the 3D inverse depth position deviation $\delta \psi^{l_{m}}$ can be marginalized out by using the left null space of the Jacobian matrix ${\bar{J}}_{\psi }$. The left null space can be calculated by QR decomposition, and we denote it as $N_{Left}$. Substituting $N_{Left}$ into the Equation (28), we have

(30) $${\bar{r}}_{MSC}^{{}^{l_{m}}}(\tilde{\chi })={\hat{\bar{r}}}_{MSC}^{{}^{l_{m}}}(\hat{\tilde{\chi }},{\hat{\psi }}^{l_{m}})+{\bar{J}}_{\tilde{\chi }}^{MSC}\delta \tilde{\chi }$$

where ${\hat{\bar{r}}}_{MSC}^{{}^{l_{m}}}(\hat{\tilde{\chi }},{\hat{\psi }}^{l_{m}})=N_{Left}{\hat{\bar{r}}}^{l_{m}}(\hat{\tilde{\chi }},{\hat{\psi }}^{l_{m}})$, ${\bar{J}}_{\tilde{\chi }}^{MSC}=N_{Left}{\bar{J}}_{\tilde{\chi }}$, and $N_{Left}{\bar{J}}_{\psi }=0$. Equation (29) is the well-known MSC factor [12,15]. It can be found that the Equation (29) is independent of the 3D inverse depth position $\delta \psi^{l_{m}}$.

By combining the last prior residual function Equation (17), the linearized and normalized pre-integration residual function in Equation (14) of the first pre-integration in the sliding window, and all of the MSC factors, we can construct the equation Equation (21). Compared with the traditional method (e.g., [8,18]), the dimension of submatrix C of our method decreases to 6 + 3 + 6 (corresponding to the first IMU pose, velocity, and biases in our implementation). We can compute the inversed matrix of C in an effective and stable manner.

## 5. Results and Discussion

We evaluated the proposed hybrid sliding window optimizer on the publicly available EuRoC datasets using a commercial laptop computer (Lenovo ThinkPad T470p, Intel i7-7700HQ, 2.8GHz). The EuRoC visual-inertial datasets were collected onboard by an MAV flying in two Vicon covered rooms and a large industrial machine hall. The datasets contain synchronized stereo image sequences, IMU measurements, and accurate ground truth [40]. All sequences were classified as easy, medium, and difficult levels according to texture, illumination, fast/slow motion, and motion blur [40]. In our data processing, we used only the left image sequence (i.e., monocular). The original publicly available ORB-SLAM has three main threads: the tracking thread, the local mapping thread, and the loop closing thread. We implemented the HSWO based on the local mapping thread, and the loop closing thread was removed. The iterative linear solver of the nonlinear VINS problem requires a good initial value to achieve a rapid rate of convergence. Hence, a good initialization for the VINS problem is necessary. In this paper, we followed the method proposed by Huang et al. [31]. A visual-inertial bundle adjustment is performed immediately after visual-inertial initialization.

We used EVO [41], a third-party evaluation software, to analyze the trajectory calculated by the HSWO. EVO aligned the trajectory with the ground truth via Umeyama’s method and then provided the RMSE of the absolute metric position deviation. Because the threads of ORB-SLAM run in parallel with other tasks of the operating system, some randomness will be introduced into the results [3]. Our implementation also inherited this property; therefore, we provide the median value of the RMSEs of five runs just as reference [3] did.

There are different choices for the size of the sliding window *N* and the size of the mature area *M*, which affects the results significantly. In this paper, we evaluated and optimize the performance of the HSWO by changing the size of *N* and *M*. In addition, the “MapPointFusion()” function in the ORB-SLAM is in charge of fusing the same map point (if available) in the scene, which may introduce some local loop closures and large loop closures. Those potential loop closures may interfere with the evaluation results and cause unfair comparison. Therefore, we adopt three methods to remove them: (a) Do not fuse the two map points if the map point with large ID has been marginalized; (b) Allow a map point to add new observation only if the new observation is within the growing region; (c) Limit the size of the Fixed basis *F*.

In the EuRoC datasets, the dataset labeled with *MH_*03*_medium* is the longest and contains fast motion that motivates the IMU. So, we analyzed the influence of changing *N*, *M*, and *F* in the HSWO by using this representative dataset. The parameter tuning results are listed in Table 1.

According to Table 1, we have

- The accuracy of the system will be improved if increase *N*, which is because more information is contained;
- By comparing *N*15-*M*10-*F*15 with *N*15-*M*10-*F*00, we can find that the accuracy will degrade dramatically if the fixed basis is removed, which is because the information in the fixed basis is omitted;
- By comparing *N*15-*M*10-*F*15 with *N*15-*M*05-*F*15, or *N*20-*M*10-*F*20 with *N*20-*M*05-*F*20, we can find that the accuracy will degrade if the value of *M* is too small, in which case the fixed basis has more weight than the prior factor, and directly fixing the pose of the keyframe in the fixed basis makes it more sensitive to the linearization error;
- By comparing *N*20-*M*10-*F*20 and *N*20-*M*15-*F*20, we can find that the accuracy of the system will also degrade if the value of *M* is too large.

Theoretically, the prior factor has one-order accuracy, the keyframe nodes related with the prior factor still have the chance to be updated. However, the linearization error of the prior factor will increase if *M* is too large, because the keyframe nodes with larger ID are estimated less times than the ones with smaller ID. For making the linearization error less and giving the keyframe nodes with large ID more freedom (i.e., making the keyframe nodes with large ID not related with the prior factor), we set *N* = 20, *M* = 10 in the following data processing.

An array of publicly available VINS pipelines (MSCKF, OKVIS, ROVIO, VINS-Mono, etc.) have been evaluated on various hardware configurations [42]. They performed sim3 trajectory alignment to the ground truth and computed the root-mean-square error (RMSE) of the absolute position over the aligned trajectory. All results are listed as a table, and we used the results evaluated on a common mobile workstation (Lenovo ThinkPad W540) for comparison. The accuracy of VI-ORB-SLAM proposed in [4] was also evaluated with the EuRoC dataset, and all metric position RMSEs are shown in the same table. Because our implementation includes no full visual-inertial bundle adjustment, we selected the results labeled as “NO full BA” for comparison. We also directly used the results reported for the VI-DSO proposed by Von Stumberg [43]. All of the results are listed in Table 2, “HSWO (Limited)” means that we removed potential local loop closures by using the three methods mentioned above, and the maximum of the size of the fixed basis *F* is set as 20. “HSWO” means that we keep the original “MapPointFusion()”, the map point will be treated as a new map point after fusion with other map point, which will cause reuse of visual measurements. Note that many details of these VINS pipelines, e.g., the data association of the front end, setting of optimizers, and trajectory saved, are different. For those reasons, the purpose of the comparison is not to show which VINS pipeline is better, but to demonstrate that our implementation can also achieve high-accuracy results by using the results provided by others VINS pipelines as a third-party reference standard.

The full trajectory contains the poses of every frame, not only the keyframe. The original ORB-SLAM recovered this trajectory from the relative pose and the reference keyframe of every frame, and we follow the same method in this paper. In most cases, the accuracy of the full trajectory may be lower than the one of the keyframe trajectory. But, in some cases, the accuracy of full trajectory may be better, and there are two reasons for this: (a) the full trajectory is recovered from the keyframe trajectory, therefore the accuracy of two trajectory should be comparable; (b) the number of pose in the full trajectory is much larger than the one in the keyframe trajectory. So, the weight of every pose deviation will be decreased during calculating the RMSE of trajectory. Then, the weight of some big deviation will also be decreased. In this case, the RMSE of the full trajectory will be less than the RMSE of the keyframe trajectory.

VI-ORB-SLAM used a conditioning-based method for fusing the visual and inertial measurements, and the first IMU pose, velocity, and biases node are also directly fixed. Theoretically, this method is more sensitive to linearization error. However, the accuracy of the keyframe trajectory produced by VI-ORB-SLAM is quite high. The reason might be: The EuRoC datasets contain many local and large loops, the “MapPointFusion ()” function in the local mapping thread may implicitly close many local loops and some large loops, which phenomenon is also found by [44]. In VINS-mono, visual features are tracked by the KLT sparse optical flow algorithm. Both VI-DSO and ROVIO use direct method for data association. Also, they cannot achieve map point fusion like ORB-SLAM. Therefore, it cannot be proven that the conditioning-based method is sufficient for VINS problem, even if the accuracy of VI-ORB-SLAM is higher than some other VINS pipelines. In order to eliminate the influence of map point fusion, the results of the HSWO in which map point fusion has been limited are also listed in Table 2. It can be found that its accuracy slightly degrades compared with the HSWO with map point fusion. Also, compared with other VINS pipelines with no map point fusion, our implementation still achieves better performance on more than half of the EuRoC datasets. Note that the VI-ORB-SLAM cannot process the *V1_03_ difficult* dataset because the movement has exceeded the limit for the monocular system [4]. In our implementation, IMU measurements are used to reckon the frame pose during tracking lost epoch, and a reprojection method is then used to perform feature matching, which makes it possible to process the *V1_03_ difficult* dataset. However, our implementation cannot process the *V2_03_ difficult* dataset if the map point fusion function is limited using the methods mentioned above, even if an approximate frame pose has been provided by integrating the IMU measurements, our implementation still cannot find suitable map points for matching.

Based on all of the above analysis, we claim that:

(a) The accuracy of the proposed HSWO will degrade if the size of mature region *M* is too small, in which case, the performance of HSWO tends to the conditioning-based method.
(b) The accuracy of HSWO will degrade if the fixed basis is removed, in this case, the HSWO degenerates into the prior-based method, and the map point whose tracking length is larger than the sliding window cannot be used effectively.
(c) We need to select a suitable value for *M* to balance the linearization error within the fixed basis and the prior factor. For our implementation, we set *N* = 20, and *M* = 10.
(d) Compared with the results provided by other VINS pipelines, the accuracy of our results is competitive.

**Figure 2:**
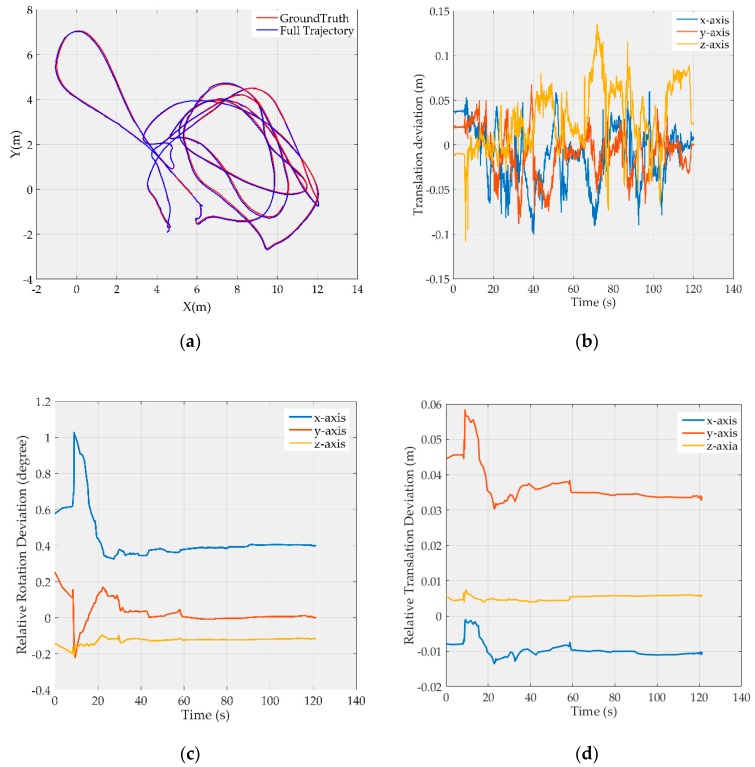
Results for the *MH_03_medium* image sequence after visual-inertial initialization: (**a**) Full trajectory after visual-inertial initialization; (**b**) Translation deviation with respect to the ground truth; (**c**) Rotation deviation of camera-to-IMU transformation; (**d**) Translation deviation of camera-to-IMU transformation.

**Figure 3:**
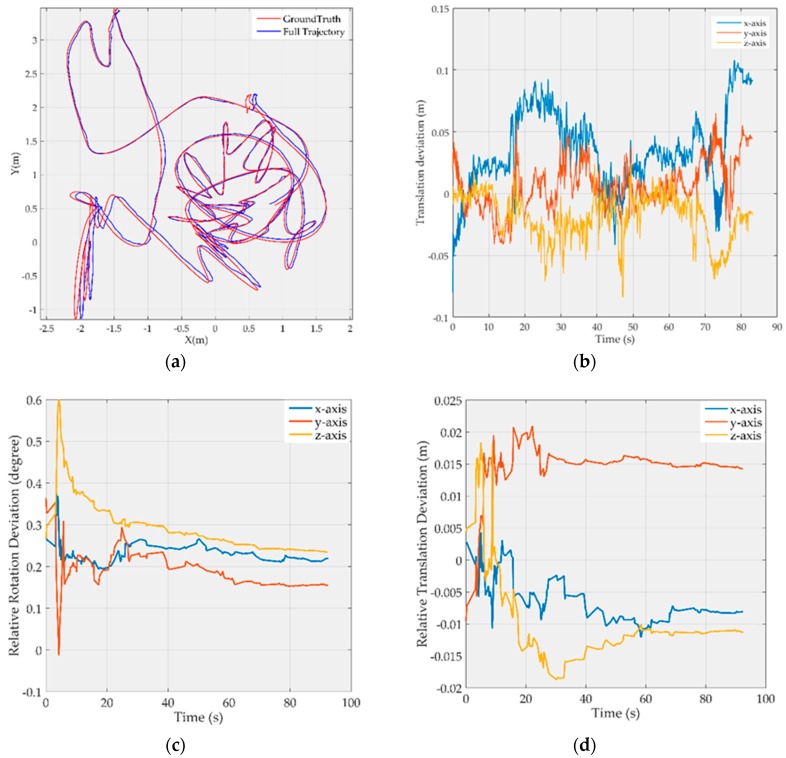
Results for the *V1_03_difficult* image sequence after visual-inertial initialization: (**a**) Full trajectory after visual-inertial initialization; (**b**) Translation deviation with respect to the ground truth; (**c**) Rotation deviation of camera-to-IMU transformation; (**d**) Translation deviation of camera-to-IMU transformation.

Due to limited space, Figure 2 and Figure 3 show only the results on *MH_03_medium* and *V1_03_difficult*, respectively, where the map point fusion function has been limited. The full trajectories are aligned with the ground truth and shown in Figure 2a and Figure 3a, respectively; the translation deviations with respect to the ground truth are shown in Figure 2b and Figure 3b, respectively, and most of the deviations are less than 10 cm; the rotation deviations of camera-to-IMU transformation are shown in the Figure 2c and Figure 3c, respectively, and the translation deviations of camera-to-IMU transformation are shown in the Figure 2d and Figure 3d, respectively. It can be found that the estimation of camera-to-IMU transformation fluctuates at the start period, and then tends to be stable as time goes on. But there are still some constant biases in the estimation. This is because the resolution of the digital camera is limited, and most map points are far from the camera, in which case, small rotation biases and small translation biases in the camera-to-IMU transformation estimation will not cause sufficient parallax, and therefore cannot be observed effectively by the optimizer.

**Figure 4:**
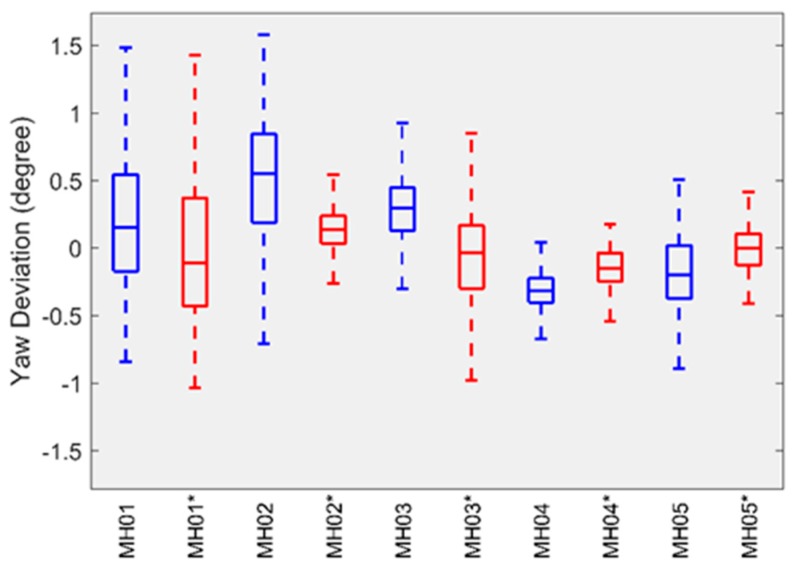
The yaw error of our implementation on Machine Hall datasets.

**Figure 5:**
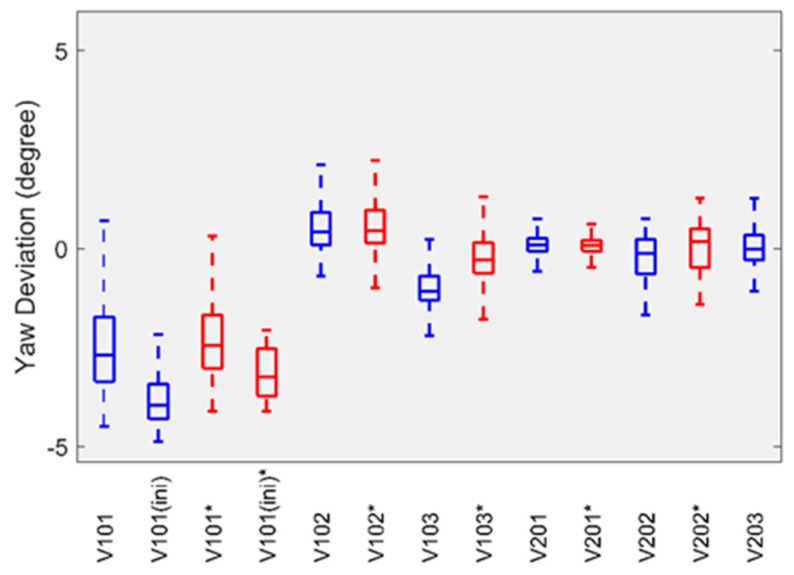
The yaw error of our implementation on Vicon room datasets.

For attitude error, we followed the evaluation method proposed by Delmerico et al. [42] and used the transformation matrix calculated by EVO to align the estimated attitude with its ground truth, and then computed the yaw error. The boxplots in Figure 4 and Figure 5 summarize the statistics of the yaw error on each sequence of the EuRoC datasets. The results provided by the HSWO (limited) are labeled with (*). The yaw error on *V1_*01*_easy* dataset is significantly larger than on the other datasets. Here we noticed that the motion on *V1_01_easy* dataset is weak during initialization, which means our implementation cannot be well initialized, even if a visual-inertial bundle adjustment is performed right after the initialization.

In the multithread framework, the optimizer has no need to operate at the frame rate. Table 3 lists the statistical results for the time consumption of the HSWO used in our implementation and the LBA (vision-only, a conditioning-based method) used in the mono ORB-SLAM [3].

The standard deviation of the time consumed by the HSWO is less than that for the LBA, which means that the HSWO’s time consumption is more stable. This better stability is a result of the fixed size of the HSWO’s sliding window, whereas the number of the keyframes used in the LBA varies according to the co-visibility between keyframes, which makes the time consumed by the LBA fluctuate. If a large number of keyframes are used in the LBA and a map point is visible in all keyframes, the second-order sparsity will disappear. In this case, the computation complexity of the LBA is cubic with the number of keyframes. Therefore, the LBA’s maximum time consumption is much larger than that of the HSWO. The mean time consumption is approximately equivalent for both methods. During the initial period, the number of keyframes is small and the co-visibility is sparse, so the LBA is much more efficient. As the map grows and the co-visibility gradually becomes denser, the time consumed by LBA will increase. Therefore, the median time consumption of the HSWO is slightly greater than that of the LBA. Based on these statistical results, we claim that the HSWO shows comparable efficiency as the LBA.

**Figure 6:**
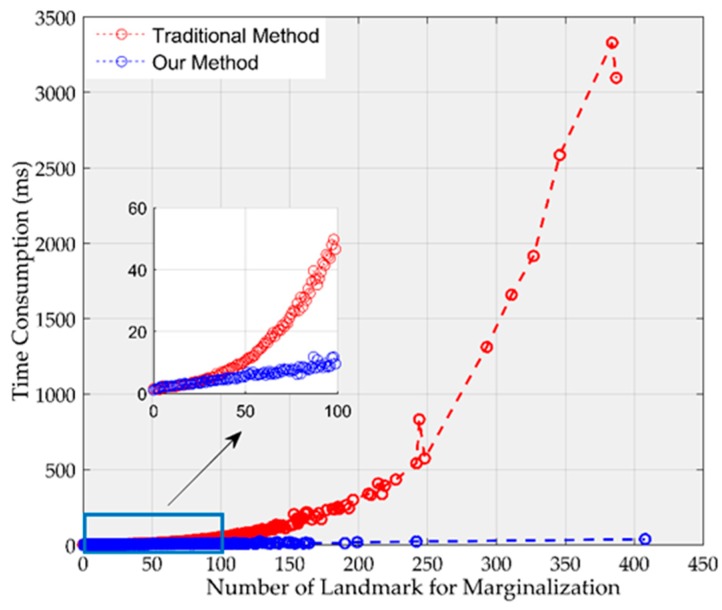
The comparison of the time consumption of our method and the traditional method.

Generally, there are 30–80 map points to be marginalized at every step. But in some cases, such map points increase to more than a hundred. In order to evaluate our marginalization method, we increased the size of mature region for making more map points marginalized. Additionally, we performed marginalization in one thread to remove randomness. Figure 6 plots the time consumed by the traditional method (e.g., [8,18]) and by our method. The time consumed by the traditional method is cubic with the number of map points used in marginalization, but the time consumed by our method is linear with the number of map points used in marginalization. Therefore, our method is much more efficient than the traditional method, especially with a large number of map points.

## 6. Conclusions

Due to the complementary sensing characteristics, low cost, and small space requirements of the visual-aided inertial navigation system, the VINS problem has become prevalent in the robotic community. In this paper, we designed a hybrid sliding window optimizer for the VINS problem, the method can effectively handle visual features whose tracking length exceeds the size of the sliding window. The sliding window was divided into two parts: the mature area and the growing area. The nodes outsides the window served as fixed basis. We marginalized a feature out only if all of its measures lay within the fixed basis and the mature area. A distributed marginalization technology was also used with significant efficiency improvement than the traditional method, and the accuracy loss was negligible. Finally, we evaluated our implementation using the open shared EuRoC datasets, and the results are competitive compared with other VINS pipelines.

## Figures and Tables

**Table 1:** The RMSEs of keyframe trajectory error with changing *N*, *M, F.*

Parameter Setting	Median of RMSEs (m)
*N*15-*M*05-*F*15	0.092
*N*15-*M*10-*F*00	0.125
*N*15-*M*10-*F*15	0.071
*N*20-*M*05-*F*20	0.068
*N*20-*M*10-*F*20	**0.055**
*N*20-*M*15-*F*20	0.063

“*Nx-My-Fz*” means *N* = *x*, *M* = *y*, and *F* = *z*.

**Table 2:** RMSEs of absolute metric position errors (m) on the EuRoC datasets.

	Cited from [42]				VI-ORB-SLAM [5]	VI-DSO [43]	HSWO		HSWO (Limited)	
	MSCKF	OKVIS	ROVIO	VINSMONO			KF Trajectory	Full Trajectory	KF Trajectory	Full Trajectory
MH01	0.42	0.16	0.21	0.27	0.075	**0.062**	**0.054**	0.048	0.078	0.075
MH02	0.45	0.22	0.25	0.12	0.084	**0.044**	**0.032**	0.036	0.072	0.057
MH03	0.23	0.24	0.25	0.13	0.087	0.117	**0.050**	0.063	0.055	**0.058**
MH04	0.37	0.34	0.49	0.23	0.217	**0.132**	**0.096**	0.099	0.169	0.216
MH05	0.48	0.47	0.52	0.35	0.082	0.121	**0.057**	0.063	0.139	**0.119**
V101	0.34	0.09	0.10	0.07	**0.027**	0.059	0.039	0.035	0.034	**0.037**
V102	0.20	0.20	0.10	0.10	**0.028**	0.067	0.032	0.036	0.042	**0.050**
V103	0.67	0.24	0.14	0.13	-	0.096	**0.054**	0.057	0.055	**0.045**
V201	0.10	0.13	0.12	0.08	**0.032**	**0.040**	0.033	0.027	0.036	0.042
V202	0.16	0.16	0.14	0.08	0.041	0.062	**0.040**	0.027	0.043	**0.029**
V203	1.13	0.29	0.14	0.21	**0.074**	0.174	0.083	0.072	-	-

The keyframe trajectory with highest accuracy is highlighted in bold and black; The best performance among the VINS pipelines, except for VI-ORB-SLAM and HSWO, is also highlighted in bold.

**Table 3:** Time consumption of the HSWO and LBA method.

METHOD	MEDIAN (ms)	MEAN (ms)	MAX (ms)	STD (ms)
HSWO	189	219	507	82
LBA	163	216	1202	189
